# Rv3787c Influences Growth Dynamics of *Mycobacterium tuberculosis*

**DOI:** 10.3390/biology15181666

**Published:** 2026-09-20

**Authors:** María Mercedes Bigi, Rosana Valeria Rocha, Elizabeth Andrea García, Federico Carlos Blanco, Fabiana Bigi

**Affiliations:** 1 Instituto de Investigaciones Biomédicas (INBIOMED), CONICET-Universidad de Buenos Aires, Buenos Aires C1121ABG, Argentina; mbigi@fmed.uba.ar; 2 Instituto de Agrobiotecnología y Biología Molecular (IABIMO), INTA-CONICET, Hurlingham 1686, Argentina; rocha.rosana@inta.gob.ar (R.V.R.); garcia.elizabeth@inta.gob.ar (E.A.G.); 3 Instituto de Biotecnología, CICVyA, Instituto Nacional de Tecnología Agropecuaria, N. Repetto and de los Reseros, Hurlingham, Buenos Aires 1686, Argentina

**Keywords:** *Mycobacterium tuberculosis*, Rv3787c, methyltransferase, THP-1

## Abstract

Rv3787c is a putative S-adenosylmethionine (SAM)-dependent methyltransferase from *Mycobacterium tuberculosis*, the main agent of human tuberculosis. Researchers identified a conserved non-synonymous polymorphism within the highly transmissible Latin American–Mediterranean lineage, suggesting that Rv3787c may play a role in host–pathogen interactions. In this study, we phenotypically characterized an *M. tuberculosis* H37Rv mutant lacking *Rv3787c* and evaluated the complementation of this mutant with strains carrying either the wild-type or mutant alleles of *Rv3787c*. The mutant strain showed no significant growth impairment under stress conditions induced by SDS or hydrogen peroxide, and it exhibited phthiocerol dimycocerosate (PDIM) and mycolic acid profiles comparable to those of the wild-type strain. Interestingly, following prolonged hypoxia and subsequent re-exposure to aerated conditions, the mutant resumed exponential growth significantly earlier than both the wild-type and complemented strains. In a human macrophage infection model, the mutant maintained persistence levels comparable to those of the wild-type and complemented strains; however, colonies were visible earlier in macrophages infected with the mutant strain. These findings suggest that while Rv3787c is not essential for intracellular survival or replication, it may influence the growth dynamics of *M. tuberculosis* under specific stress conditions.

## Simple Summary

Tuberculosis, caused by the bacterium *Mycobacterium tuberculosis*, remains one of the world’s deadliest infectious diseases. Identifying which bacterial genes aid survival and transmission could reveal new therapeutic targets. In this study, we studied *Rv3787c*, which belongs to a family of genes encoding enzymes involved in modifying other internal bacterial molecules. Previous research identified a variation in this gene within *M. tuberculosis* strains that spread easily among humans, suggesting that *Rv3787c* helps the bacterium interact with the host. Bacteria lacking the *Rv3787c* gene grew normally under most stress conditions and produced surface fatty molecules comparable to those of wild-type bacteria. However, after enduring prolonged oxygen deprivation—a condition mimicking the host environment—the mutant bacteria resumed growth faster than normal strains once oxygen returned. We also detected these mutant bacteria slightly earlier when infecting human immune cells, although they survived and multiplied inside these cells as effectively as normal bacteria. Overall, our results demonstrate that *Rv3787c* is not essential for intracellular survival, but it may delay the bacterium’s ability to restart growth after stressful conditions. This suggests the gene acts as a kind of “brake” on bacterial growth under specific circumstances.

## 1. Introduction

*Mycobacterium tuberculosis* is the principal causative agent of human tuberculosis (TB). This pathogen belongs to the *M. tuberculosis* complex (MTBC), a taxonomic group comprising species that cause TB in mammals. The *M. tuberculosis* genome encodes approximately 4000 proteins, and researchers have established physiological roles or infection relevance for many of them. For instance, high-throughput transposon mutagenesis enabled researchers to identify more than 190 genes essential for *M. tuberculosis* survival and replication in a murine model [1]. Despite such advances, researchers still poorly understand many genes, predicting their functions solely from conserved domains—or lacking functional annotation altogether.

One such protein is Rv3787c, a putative S-adenosylmethionine (SAM)-dependent methyltransferase. MTBC species as well as non-tuberculous mycobacteria, including *M. marinum* and *M. smegmatis*, conserve Rv3787c. The highly successful, multidrug-resistant outbreak strain M—which belongs to the Latin American–Mediterranean (LAM) lineage—harbors a non-synonymous mutation (A291V) in Rv3787c. The LAM lineage represents one of the major phylogenetic groups of *M. tuberculosis* and is particularly prevalent in South America, including Argentina [2]. Notably, all high-fitness M-family isolates conserve this mutation in *Rv3787c* [3], suggesting that the variation plays a potential role in bacterial adaptation or virulence.

SAM-dependent methyltransferases constitute a critical enzyme family that employs S-adenosylmethionine as a methyl donor to regulate diverse biological processes, including DNA, RNA, and protein methylation, as well as virulence, antibiotic resistance, and stress responses. The *Rv3787c* gene lies adjacent to *Rv3786c*, a gene encoding a poorly characterized protein containing conserved WcaA glycosyltransferase and peptidase M23 domains. The genomic organization of these genes suggests possible co-transcription, although researchers have not yet demonstrated this experimentally. Given the predicted domains of *Rv3786c*, their genomic proximity suggests that Rv3787c and Rv3786c may functionally participate in cell envelope metabolism.

Dejesus and collaborators [4] reported *Rv3787c* as one of more than 300 genes whose disruption by transposon mutagenesis confers a growth advantage in *M. tuberculosis*. Researchers have associated altered methyltransferase activity with accelerated growth in mycobacteria. Specifically, recent observations in clinical *M. tuberculosis* isolates revealed a link between an anomalous methylation profile in DNA methyltransferase C (MamC) and faster growth, suggesting that changes in DNA methylation influence bacterial growth dynamics [5].

In a previous study, we generated an *Rv3787c*-deleted mutant in the reference strain *M. tuberculosis* H37Rv [6]. Lipids isolated from this mutant—unlike those from the parental wild-type strain—inhibited the H37Rv-induced T-cell response in vitro in human peripheral blood mononuclear cells (PBMCs) [6].

Taken together, these previous findings—including the enhanced in vitro growth associated with *Rv3787c* transposon disruption, the altered immunomodulatory properties of lipids from the *Rv3787c* mutant, and the conservation of a specific *Rv3787c* mutation among high-fitness strains—suggest that Rv3787c influences *M. tuberculosis* fitness and host–pathogen interactions. To investigate this hypothesis, we assessed the replication capacity of the *Rv3787c* knockout mutant under various in vitro and ex vivo conditions and evaluated its response to different environmental stresses. Finally, we investigated whether introducing the *Rv3787c* allele present in M strain could complement the phenotypes observed in the knockout mutant.

## 2. Materials and Methods

### 2.1. Strains, Complementation, and Culture Conditions

#### 2.1.1. Strains

In this study, we used the reference strain *M. tuberculosis* H37Rv, a mutant derivative lacking the *Rv3787c* gene (hereafter referred to as ΔRv3787c) generated previously by recombineering [6], and two complemented strains, ΔRv3787c::Rv3787c^WT and ΔRv3787c::Rv3787c^M (this study).

#### 2.1.2. Mycobacterial Growth Conditions

*M. tuberculosis* strains were cultivated in Middlebrook 7H9 or 7H10 medium (BD Diagnostic Systems, Sparks, MD, USA) supplemented with 0.4% glycerol (Millipore Sigma, Burlington, MA, USA), 0.5% bovine albumin (Fraction V, Millipore Sigma, Burlington, MA, USA), 0.4% glucose (Biopack, CABA, Buenos Aires, Argentina), and 0.05% Tween 80 (Millipore Sigma, Burlington, MA, USA) in T25 tissue culture flasks under agitation.

For the growth curve analysis, two conditions were evaluated: (i) Liquid cultures were inoculated in T25 flasks using exponential-phase starter cultures (10-day-old cultures), adjusted to an initial optical density at 600 nm (OD_600nm_) of 0.001, and incubated at 37 °C with agitation. Growth was monitored periodically by measuring the OD_600nm_. (ii) For the second condition, starter cultures were grown under hypoxia for 20 days using the Wayne model [7]. Briefly, aerated cultures (OD_600nm_ = 0.4–0.6) were diluted to an OD_600nm_ of 0.05 and transferred into 60 mL flat-bottom glass tubes filled to 80% of their total volume and sealed with screw caps with O-rings. The tubes were placed on a magnetic stirrer, and oxygen depletion was monitored using a methylene blue (1.5 µg/mL) indicator control (Figure S1). After 20 days of hypoxic incubation, the cultures were diluted 1:3 in triplicate, ensuring that approximately 75% of the tube volume remained unfilled to allow oxygen re-exposure, and incubated at 37 °C with magnetic stirring. Growth was monitored periodically by measuring the OD_600_.

#### 2.1.3. Strain Complementation

The full open reading frame of the *Rv3787c* gene was PCR-amplified from the strain H37Rv and the M strain using primers described in Table S1 [3]. The amplified fragments were cloned into the *Nde*I and *Hind*III restriction sites of the pVV16 plasmid downstream of the *hsp60* promoter, as described in detail in Supplementary materials and Methods Section. The resulting recombinant plasmids were introduced into the ΔRv3787c strain by electroporation.

Briefly, 50 mL of an exponentially growing ΔRv3787c culture was harvested by centrifugation at 2000 *g* for 12 min. The pellet was resuspended in 45 mL of 10% glycerol prepared in ultrapure water and centrifuged again to collect the cells. This washing step was repeated four times. The final mycobacterial pellet was resuspended in 1 mL of 10% glycerol.

For electroporation, 1 µg of the recombinant plasmids was mixed with 200 µL of competent mycobacterial cells and transferred to a 0.2 cm electroporation cuvette. Electroporation was performed using a single pulse applied at 2.5 kV, 1000 Ω, and 25 µF. Immediately after electroporation, the cell mixtures were transferred to tubes containing 5 mL of complete 7H9 medium and incubated overnight at 37 °C with shaking. The cultures were then plated on complete 7H10 medium supplemented with kanamycin (20 µg/mL). Recombinant colonies were observed after 20–25 days of incubation at 37 °C.

The presence of the plasmids in the complemented strains was confirmed by PCR amplification of *Rv3787c*, as described in the Supplementary Materials and Methods Section. The resulting complemented strains were designated ΔRv3787c::Rv3787c^WT and ΔRv3787c::Rv3787c^M. ΔRv3787c was maintained in medium supplemented with hygromycin (50 µg/mL), whereas the complemented strains were grown in medium containing both hygromycin (50 µg/mL) and kanamycin (20 µg/mL).

#### 2.1.4. Stress Susceptibility Assays

Susceptibility to SDS (10%, Promega Corporation, Madison, WI, USA) and H_2_O_2_ (6%) was assessed by disc diffusion. Briefly, bacterial suspensions containing 10^7^ colony forming units (CFU) were plated on Middlebrook 7H10 agar, and sterile discs loaded with 10 µL of each compound were placed at the center. Zones of inhibition were measured after two to three weeks of incubation at 37 °C.

### 2.2. RT-PCR/qPCR

Total RNA was extracted from *M. tuberculosis* strains following the method described by Santangelo and collaborators [8]. Briefly, 50 mL of bacterial culture was harvested by centrifugation, and the cell pellets were resuspended in 0.5 mL of TRIzol™ Reagent (Invitrogen, Thermo Fisher Scientific, Waltham, MA, USA) in 1.5 mL screw-cap tubes containing zirconia beads. The cells were disrupted using a Precellys homogenizer (Bertin Technologies, Montigny-le-Bretonneux, France), followed by centrifugation at 9000 *g* for 5 min at room temperature. The supernatants were transferred to RNase-free 1.5 mL tubes and extracted twice with 200 μL of chloroform. The aqueous phases containing nucleic acids were transferred to clean tubes, and ammonium acetate (5 M; 10% of the aqueous phase volume) and 0.7 volumes of isopropanol were added. The samples were incubated overnight at −80 °C. RNA was pelleted by centrifugation at 10,000 *g* for 30 min at 4 °C, and the pellets were washed with 70% ethanol. The RNA samples were further purified using the Monarch RNA Cleanup Kit (New England Biolabs, Ipswich, MA, USA) according to the manufacturer’s instructions and quantified using a NanoDrop spectrophotometer (Thermo Fisher Scientific, Wilmington, DE, USA). Residual genomic DNA was removed by treating 2 μg of RNA from each sample with DNase I (Ambion, Life Technologies Invitrogen, CA, USA). Samples were incubated for 30 min at 37 °C in 1× DNase reaction buffer with 2 μL of DNase I. An additional 2 μL of DNase I was then added, and samples were incubated again for 30 min at 37 °C. The enzyme was inactivated by the addition of EDTA, followed by incubation at 65 °C for 15 min.

First-strand cDNA was synthesized from DNase-treated RNA using M-MLV Reverse Transcriptase (Promega Corporation, Madison, WI, USA) and random primers (Invitrogen, Thermo Fisher Scientific, Waltham, MA, USA). Briefly, 1 μg of total RNA (20–25 ng) was mixed with random primers (60 µg) and nuclease-free water to a final volume of ≤13.38 μL. The RNA-primer mixture was incubated at 70 °C for 5 min and immediately cooled on ice. Subsequently, 5 μL of 5× M-MLV RT reaction buffer, 1.25 μL of 10 mM dNTP mix (dATP, dCTP, dGTP, and dTTP), and 200 U of M-MLV Reverse Transcriptase were added to the mixture. The reaction volume was adjusted to 25 μL with nuclease-free water, and reverse transcription was performed at 37 °C for 60 min. Reactions without M-MLV RT were included as no-RT controls. Quantitative PCR (qPCR) amplifications were performed using 1 µL of cDNA as a template on a StepOne Plus Real-Time PCR System (Applied Biosystems, Foster City, CA, USA) in technical duplicates, using the SsoFast EvaGreen Supermix (BIO-RAD, Hercules, CA, USA). The cycling conditions were as follows: an initial incubation at 95 °C for 5 min, followed by 40 cycles of 60 °C for 30 s and 72 °C for 30 s. The *sigA* gene was used as a reference, and H37Rv served as the calibrator sample.

### 2.3. Lipid Analysis

*M. tuberculosis* strains were grown to the exponential phase (OD_600nm_ = 0.3–0.6) as described above. Total lipids were extracted following the method of Jackson and collaborators [9]. Briefly, 50 mL of each culture grown until exponential phase was harvested by centrifugation at 9000 *g*, resuspended in 1 mL of ultrapure water in pre-weighed 2 mL tubes, and heat-inactivated at 80 °C for 30 min. The suspensions were then lyophilized, and the dry pellets were weighed to determine the dry weight of each culture using an analytical balance.

Cell pellets were subjected to sequential overnight lipid extractions. Initially, lipids were extracted with chloroform:methanol (1:2, *v*/*v*), followed by two additional overnight extractions with chloroform:methanol (2:1, *v*/*v*). A final extraction step was performed using chloroform:methanol:water (3:47:48, *v*/*v*/*v*). Vacuum centrifugation was used to remove the organic solvent, and lipids were weighed using an analytical balance. Total extractable lipids were subsequently resuspended in chloroform:methanol (2:1, *v*/*v*). The lipids were resuspended in a defined volume of solvent to achieve the same concentration across all samples.

The volume was estimated based on the dry weight of the inactivated, lyophilized mycobacteria. Mycolic acid methyl esters (MAMEs) were derived from extractable lipids as follows: total lipids or delipidated bacterial cells (from 50 mL cultures) were resuspended in 1 mL of tetramethylammonium hydroxide 15% and saponified overnight at 100 °C. Subsequently, 1.5 mL of dichloromethane was added, and the mixture was agitated at room temperature for 4 h to allow methylation of fatty acids. The samples were then centrifuged, and the upper aqueous phase was discarded. The lower organic phase was washed three times with water, after which the organic phase was evaporated using a vacuum centrifuge dryer. The resulting pellet was resuspended in diethyl ether and sonicated in an ultrasonic water bath for 5 min. The samples were centrifuged, transferred to a new tube, and dried under a hood to obtain the ether phase. Finally, lipids were resuspended in dichloromethane.

Lipids were resolved by thin-layer chromatography (TLC, MilliporeSigma, Burlington, MA, USA) on silica gel 60F_254_ plates, loading equal amounts per lane (≈300 µg). The TLC plates were developed using petroleum ether: ethyl acetate (98:2) for phthiocerol dimycocerosate (PDIM) or η-hexane:ethyl acetate (95:5 *v*/*v*) (three sequential developments) for MAMEs. Lipids were visualized by spraying with a CuSO_4_-phosphoric acid solution, followed by heating with a hot air gun. All experiments were performed in three independent biological replicates.

### 2.4. Macrophage Infections

The human acute monocytic leukemia cell line THP-1 was resuspended in Roswell Park Memorial Institute (RPMI) 1640 medium supplemented with 10% fetal bovine serum (FBS) and 50 nM phorbol 12-myristate 13-acetate (PMA, Millipore Sigma, Burlington, MA, USA), and seeded into a 96-well plate at a density of 1 × 10^5^ cells per well to induce differentiation into macrophage-like cells. After 24 h, adherent monolayers were washed with phosphate-buffered saline (PBS) and infected with *M. tuberculosis* strains at a multiplicity of infection (MOI) of 1:1. Mycobacterial suspensions were prepared in RPMI without FBS.

Following a 4 h incubation, the culture supernatant was removed, and monolayers were washed with PBS to eliminate extracellular bacteria. Fresh RPMI (300 µL) supplemented with 10% FBS and gentamicin (5 µg/mL) was then added and maintained for 72 h.

At the indicated time points, the culture medium was aspirated, and cells were washed and lysed with 100 µL of sterile water. Serial dilutions of the lysates were plated onto solid medium and incubated at 37 °C for up to five weeks for CFU determination. Colony growth was monitored throughout the incubation period, and CFUs were counted once individual colonies could be clearly distinguished.

The entire area occupied by bacterial colonies on each plate was selected as the region of interest, and the integrated intensity was quantified using ImageJ software (National Institutes of Health, Bethesda, MD, USA, https://imagej.net/, accessed on 1 September 2026). The same image acquisition and analysis settings were applied to all samples to ensure consistency among measurements. The integrated intensity values obtained for each plate were used for subsequent statistical analysis. Quantitative measurements were obtained in triplicate and used for statistical analysis.

### 2.5. Statistical Analysis

Graphs were generated using GraphPad Prism version 5 (GraphPad Software, San Diego, CA, USA). Mycobacterial growth, both in vitro and in vivo, was analyzed using a two-way analysis of variance (ANOVA) followed by Bonferroni’s post hoc test. For the disc diffusion assay, statistical significance was assessed using Student’s *t*-test. ImageJ quantification data were analyzed using two-way ANOVA. Amplification curves were analyzed using LinRegPCR (LinRegPCR 20210614) software (https://www.gear-genomics.com/rdml-tools/, accessed on 1 September 2026) to determine cycle threshold (Ct) values and individual reaction efficiencies. Relative gene expression, reported as fold change, was calculated via the efficiency-corrected ΔΔCt method, while statistical comparisons between conditions were performed using the Pair Wise Fixed Reallocation Randomization Test. All calculations were carried out within the InfoStat 1 statistical software package (https://www.infostat.com.ar/index.php?mod=page&id=34, accessed on 1 September 2026), with the fgStatistics module. Differences were considered significant at *p* < 0.05.

### 2.6. In Silico Structural Analysis of Rv3787c

The AlphaFold structural model of Rv3787c from *M. tuberculosis* H37Rv (UniProt accession P9WFH3; AlphaFold model AF-P9WFH3-F1) was retrieved from the AlphaFold Protein Structure Database. Predicted S-adenosyl-L-methionine (SAM)-binding residues (residues 131, 160, and 161) were obtained from UniProt annotations assigned by sequence similarity. Structural visualization was performed using UCSF ChimeraX v1.11 [10]. Residue A291, corresponding to the naturally occurring A291V substitution identified in the Mp strain, and the predicted SAM-binding residues were mapped onto the structural model. The effect of the A291V substitution on protein stability was evaluated using DynaMut2 [11], which predicts the change in folding free energy (ΔΔG) associated with amino acid substitutions.

## 3. Results

### 3.1. Phenotypic Characterization of ΔRv3787c

**Figure 1:**
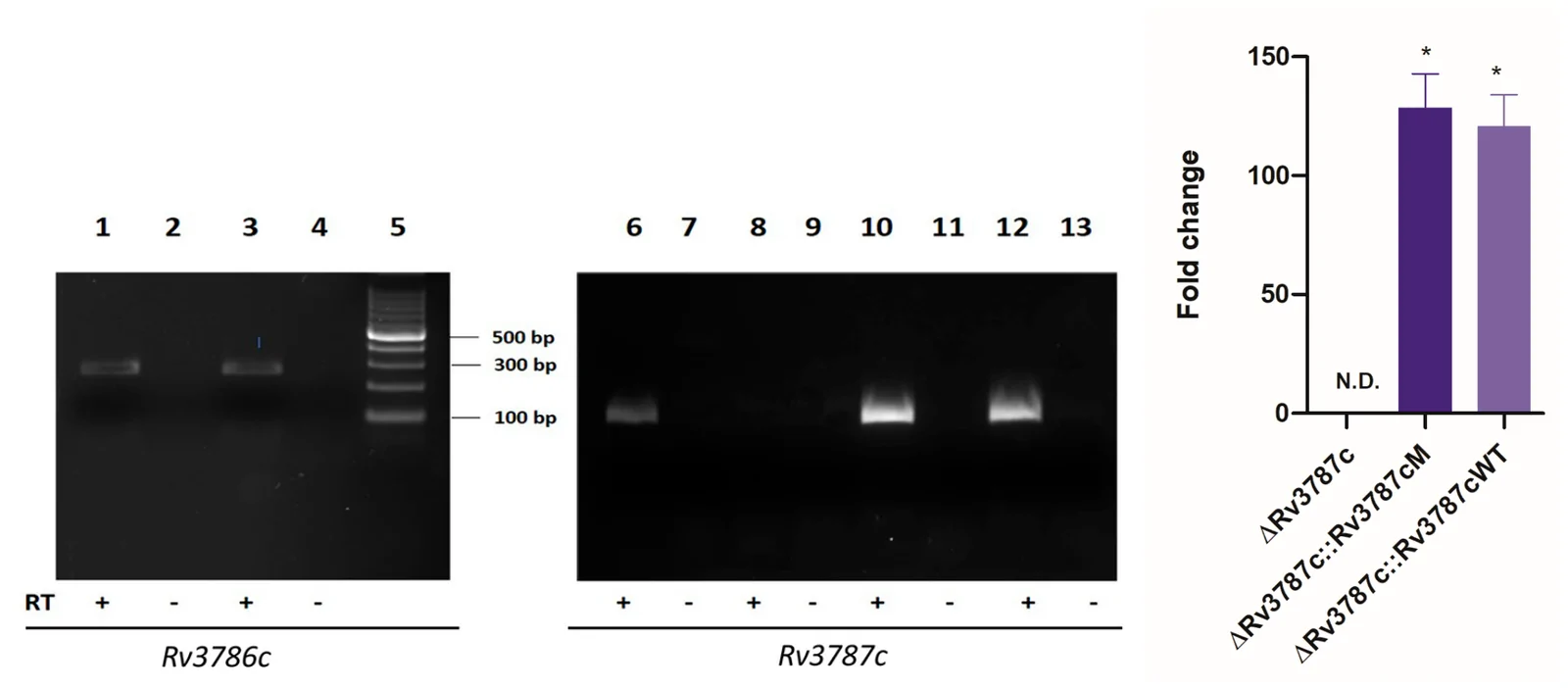
Transcriptional analysis of *Rv3787c* and *Rv3786c* in *M. tuberculosis* strains. (**Left** and **middle**) Total RNA was isolated from wild-type *M. tuberculosis* H37Rv (wild type; lanes 1, 2, 6, and 7), ΔRv3787c (lanes 3, 4, 8, and 9), and the complemented strains ΔRv3787c::Rv3787c^M (lanes 10 and 11) and ΔRv3787c::Rv3787c^M (lanes 12 and 13). Lane 5: Molecular weight marker (MWM). In lanes 2, 4, 7, 9, 11, and 13, the RT enzyme was omitted (no-RT controls). (**Right**) Relative transcript levels were quantified by real-time RT-qPCR using *sigA* as an endogenous reference and H37Rv as the calibrator sample. The Pair Wise Fixed Reallocation Randomization Test was used to assess fold-change differences between H37Rv and ΔRv3787c, ΔRv3787c::Rv3787c^M, and ΔRv3787c::Rv3787c^M strains (*p* < 0.05). These experiments were performed once. N.D.: no determined, * *p* < 0.05.

In a previous study, we generated an *Rv3787c* deletion mutant in the *M. tuberculosis* H37Rv reference strain using recombineering technology [6]. The resulting mutant strain, designated H37RvΔRv3787c (ΔRv3787c), was constructed by replacing the *Rv3787c* gene with a hygromycin resistance cassette. Since *Rv3787c* is predicted to form an operon with *Rv3786c*, insertion of the resistance marker could potentially exert polar effects on downstream *Rv3786c* transcription. To evaluate this, we performed RT-PCR (Figure 1) and RT-qPCR (Figure S2) analyses of *Rv3786c* expression in ΔRv3787c and found that *Rv3786c* expression remained unchanged in the mutant. This finding confirmed that the hygromycin cassette did not disrupt its transcription.

Complementation of ΔRv3787c with replicative plasmids carrying *Rv3787c* from H37Rv (*Rv3787cWT*) or the mutant allele from the M strain (*Rv3787cM*) restored *Rv3787c* transcription, as demonstrated by RT-PCR (Figure 1). RT-PCR also confirmed the absence of the *Rv3787c* transcript in ΔRv3787c (Figure 1). Given that the complementation plasmids carrying the different *Rv3787c* alleles contain a mycobacterial origin of replication and are therefore expected to be maintained at multiple copies, we next compared *Rv3787c* transcription levels among the strains. RT-qPCR analysis showed that both complemented strains exhibited significantly higher *Rv3787c* transcript levels than the wild-type strain (Figure 1).

The potential structural consequences of the naturally occurring A291V substitution were evaluated by performing an in silico analysis using the AlphaFold model of Rv3787c. DynaMut2 predicted a modest destabilizing effect on protein stability (ΔΔG = −1.18 kcal/mol). Mapping of the substitution onto the predicted structure showed that residue 291 is located distal to the putative SAM-binding residues (131, 160, and 161), suggesting a low probability that this substitution directly affects cofactor recognition (Figure S3).

To assess whether Rv3787c contributes to stress resistance, we compared ΔRv3787c and wild-type strains using disc diffusion assays with SDS or H_2_O_2_. No significant differences in zones of inhibition were observed between the strains (Table S2), suggesting that Rv3787c is not involved in resistance to these compounds.

### 3.2. Deletion of Rv3787c Does Not Significantly Impact in the PDIM and Mycolic Acid Profile

**Figure 2:**
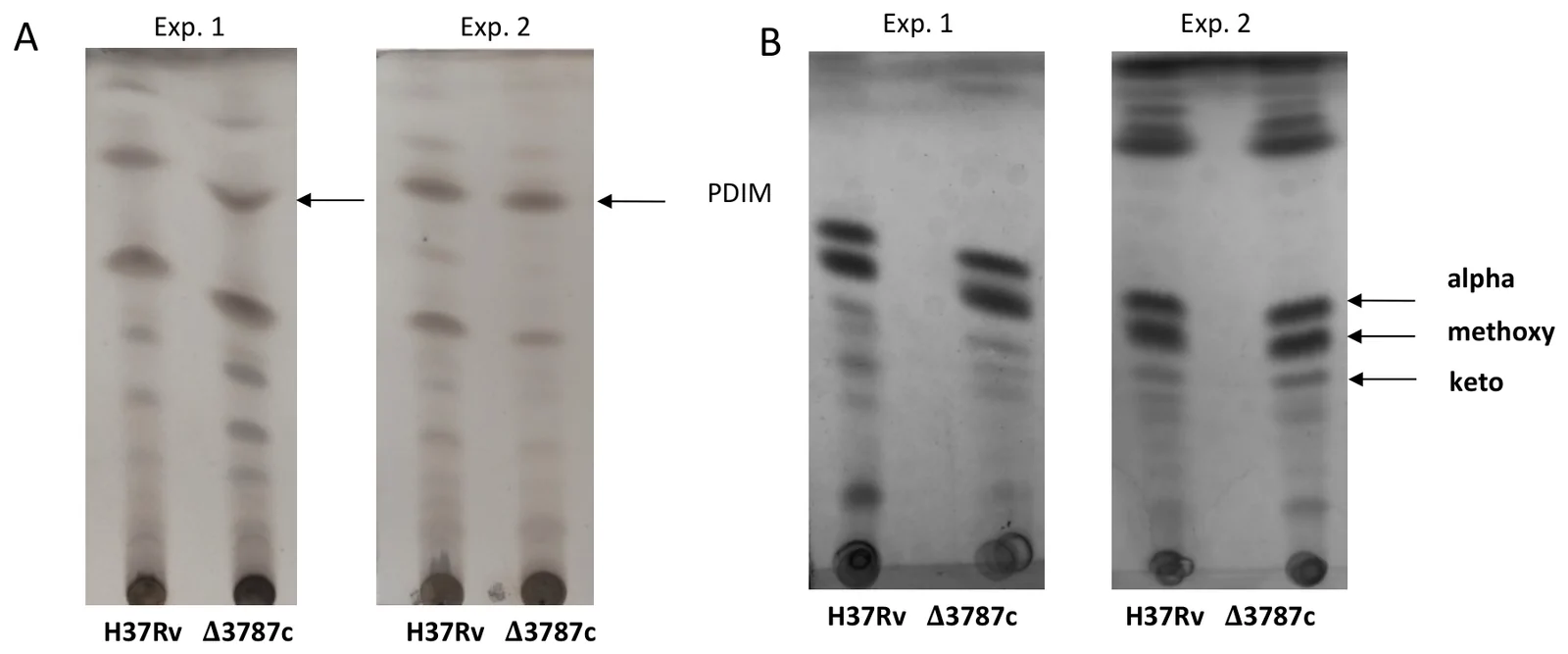
Lipid profile of *M. tuberculosis* strains. Lipids were extracted from *M. tuberculosis* strains cultured in 7H9 medium supplemented with 0.5% glycerol, 0.5% bovine albumin, 0.4% glucose, and 0.05% Tween-80 under agitation until reaching stationary phase. (**A**) Lipid extracts were analyzed by TLC using petroleum ether: ethyl acetate (98:2) to resolve PDIMs. Lipids were visualized by spraying with CuSO_4_ followed by heating. (**B**) Mycolic acid methyl esters (MAMEs) were prepared from total extractable lipids and analyzed by TLC using *n*-hexane:ethyl acetate (95:5, *v*/*v*) with three sequential developments. Lipids were visualized by spraying with CuSO_4_, followed by heating.

Given the reported role of SAM-dependent methyltransferases in PDIM and mycolic acid biosynthesis, these lipid classes were analyzed by TLC in the wild-type (WT), ΔRv3787c, and complemented strains. The lipid profiles were indistinguishable among the strains, with no substantial differences detected in either PDIM or mycolic acid composition (Figure 2).

### 3.3. Deletion of Rv3787c Enhances In Vitro Replication of M. Tuberculosis

To phenotypically characterize the ΔRv3787c, we compared its growth kinetics with those of the wild-type and complemented strains in liquid medium supplemented with ADC and Tween-80. Cultures were inoculated with bacteria previously subjected to 20 days of oxygen depletion, and re-aerated cultures were grown as described in Materials and Methods.

**Figure 3:**
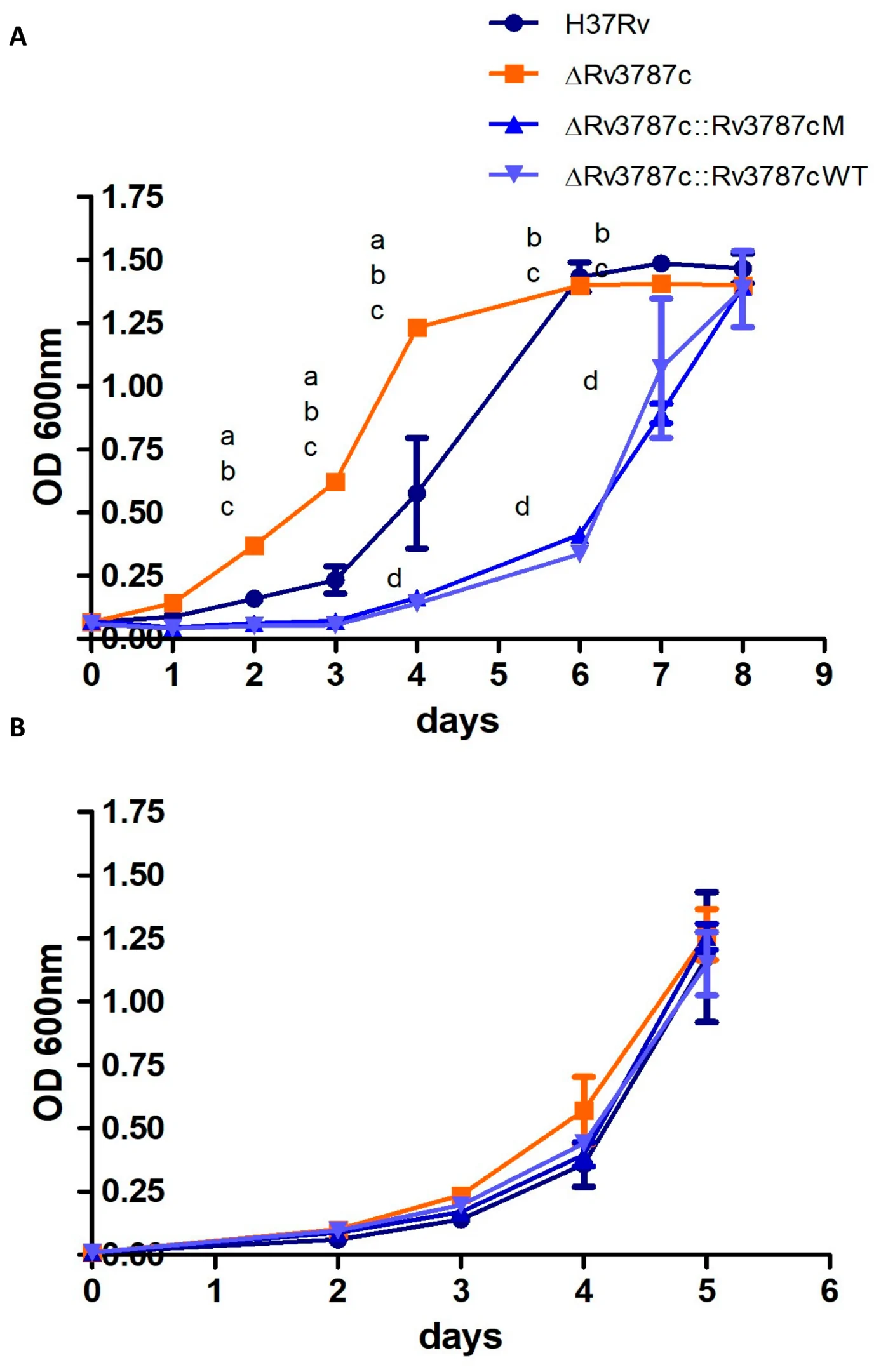
In vitro growth kinetics of *M. tuberculosis* strains. Wild-type *M. tuberculosis* H37Rv, the ΔRv3787c, and the complemented strains ΔRv3787c::Rv3787c^WT and ΔRv3787c::Rv3787c^M were inoculated from either (**A**) 20-day hypoxic stationary-phase (60-day total culture) starter cultures or (**B**) exponential-phase (7-day culture) starter cultures into Middlebrook 7H9 medium supplemented with 0.4% glycerol, 0.5% BSA, 0.4% glucose, and 0.05% Tween-80. Cultures were incubated under agitation using a magnetic stirrer (**A**) or a shaker (**B**). Growth was monitored at the indicated time points by measuring OD_600nm_. Data are representative of three independent experiments. Values represent the mean ± standard deviation (SD) of three technical replicates. Statistical analysis was performed using two-way ANOVA followed by Bonferroni’s post hoc test. Significance is indicated as follows: a, ΔRv3787c vs. H37Rv (*p* < 0.01); b, ΔRv3787c vs. ΔRv3787c::Rv3787cWT (*p* < 0.001); c, ΔRv3787c vs. ΔRv3787c::Rv3787cM (*p* < 0.001); d, H37Rv vs. ΔRv3787c::Rv3787cWT or ΔRv3787c::Rv3787cM (*p* < 0.001).

During the early phase of incubation, all strains exhibited similarly low replication rates. Thereafter, ΔRv3787c grew faster than the wild-type and complemented strains, exhibiting increased growth (Figure 3A). At various time points, ΔRv3787c showed significant differences in growth compared with both the wild-type and complemented strains; however, the specific time points at which these differences became significant varied between independent experiments.

In contrast, when inocula from exponential-phase cultures were used, all strains showed similar growth kinetics (Figure 3B). These findings suggest that the absence of Rv3787c enhances the capacity of *M. tuberculosis* to resume growth following hypoxic stress, resulting in faster bacterial recovery under post-hypoxic conditions.

### 3.4. Deletion of Rv3787c Results in Faster Growth of M. Tuberculosis Following Macrophage Infection

We next assessed the intracellular replication of the ΔRv3787c, wild-type, and complemented strains using THP-1 macrophages. Following infection, infected macrophages were maintained for the indicated periods, and bacterial loads were determined by plating serial dilutions of cell lysates on solid medium to CFUs. At each time point, CFUs were used to quantify the number of viable bacteria recovered from the infected cells. In addition, we monitored the appearance and subsequent growth of individual colonies on solid medium.

**Figure 4:**
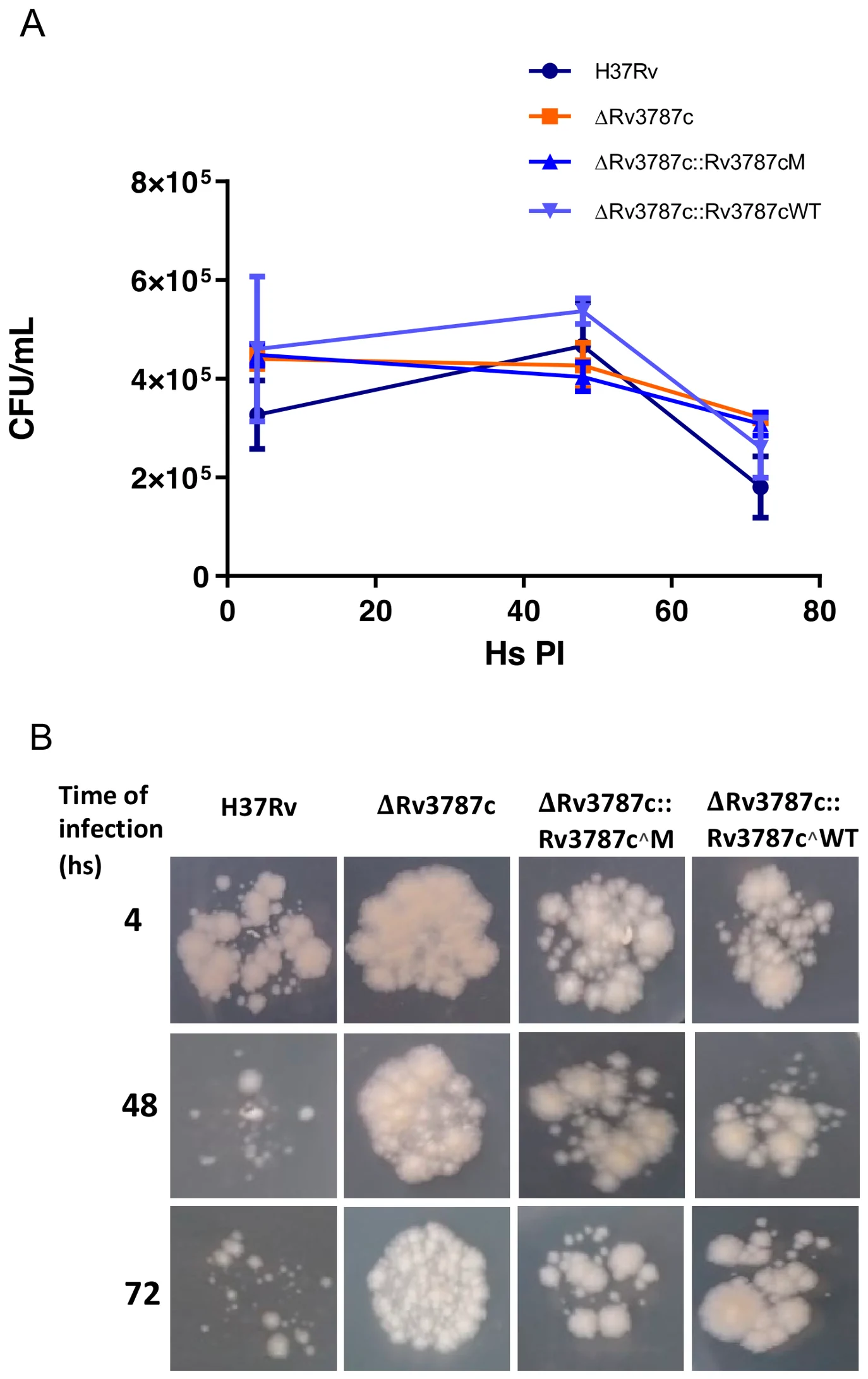

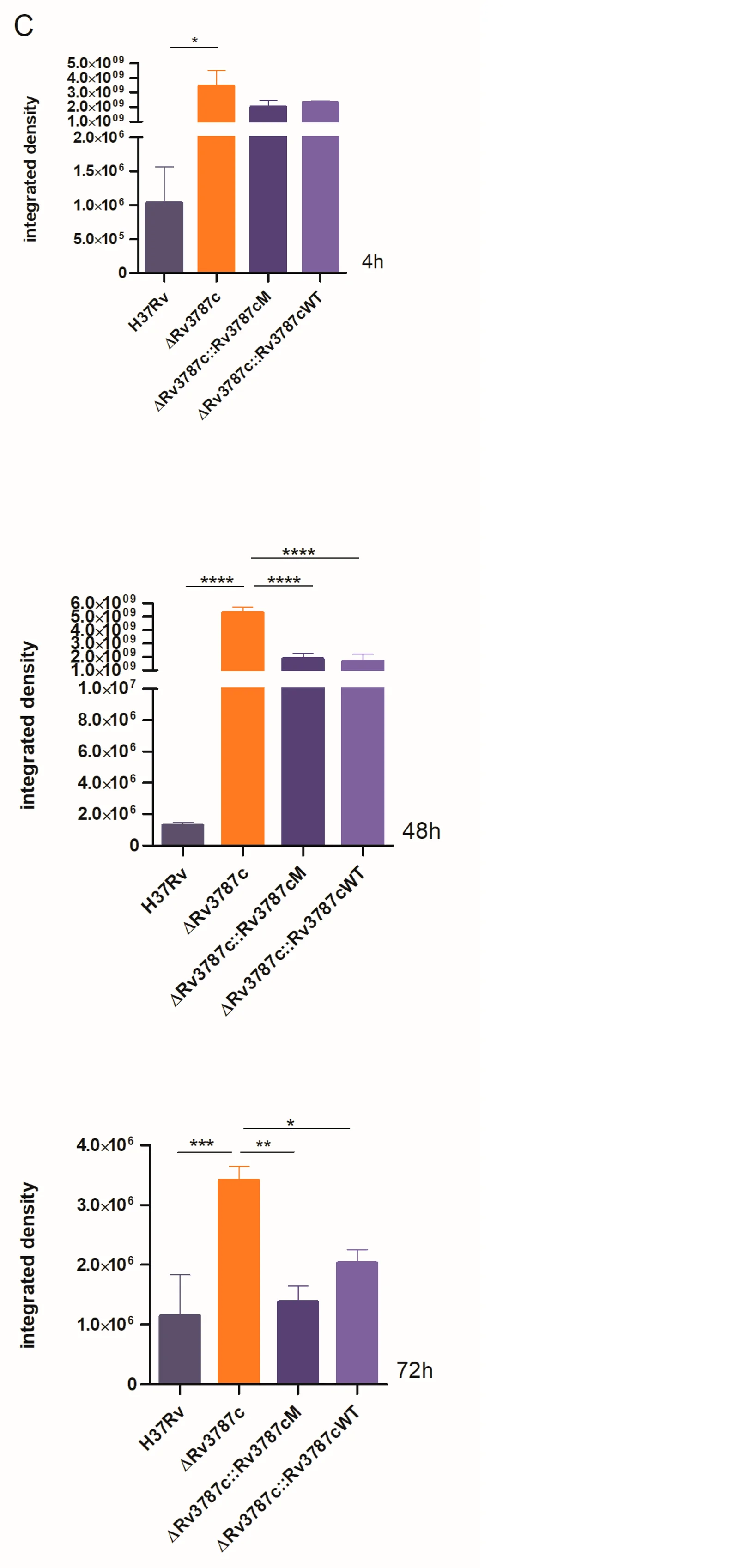
Intracellular persistence of *M. tuberculosis* strains in human macrophages. THP-1-derived macrophages were infected with wild-type *M. tuberculosis* H37Rv, the ΔRv3787c, or the complemented strains ΔRv3787c::Rv3787c^WT and ΔRv3787c::Rv3787c^M (MOI = 1:1), as described in Materials and Methods. (**A**) Bacterial survival was assessed by quantifying CFUs at 4, 48, and 72 h post-infection (hpi) following macrophage lysis. Data are representative of two independent experiments with similar results. Values represent the mean ± standard deviation (SD) of three technical replicates. Statistical analysis was performed using two-way ANOVA followed by Bonferroni’s post hoc test. Error bars represent standard deviation. (**B**) Representative images acquired 30 days after plating showing CFUs from each strain that persisted intracellularly at the time points indicated on the left side of the figure. (**C**) Colony growth was quantified from digital images using ImageJ. The integrated density of the area occupied by bacterial colonies was measured for each plate as a quantitative measure of colony growth. Measurements were performed on plates containing bacteria recovered from macrophages at the indicated time points. Data represent the mean ± standard deviation (SD) of three technical replicates. Significance was assessed by two-way ANOVA (* *p* < 0.05, ** *p* < 0.01, *** *p* < 0.001, **** *p* < 0.0001).

After 30 days of incubation, the number of CFUs recovered from THP-1 macrophages was comparable between ΔRv3787c, wild-type, and complemented strains (Figure 4A), indicating no detectable difference in bacterial burden under the conditions tested. However, inspection of plates at various time points following plating of infected macrophage lysates revealed clear differences in colony size between strains. Colonies derived from ΔRv3787c were larger than those of the wild-type and complemented strains, indicative of a faster growth rate (Figure 4B,C and Figure S4). Importantly, this difference was confined to colony size rather than colony number, indicating that the phenotype reflects differences in the rate of colony growth rather than differences in bacterial survival or recovery from macrophages. These results indicate that *Rv3787c* deletion results in a transiently faster growth phenotype upon recovery from macrophage infection, without affecting the overall number of viable bacteria recovered.

## 4. Discussion

The *M. tuberculosis* genome encodes 21 putative methyltransferases, with S-adenosylmethionine (SAM)-dependent methyltransferases representing the largest group. Although SAM-dependent methyltransferases typically catalyze methylation reactions, most functionally characterized members in *M. tuberculosis* specifically modify mycolic acids. These include MmaA1 (Rv0645c), MmaA2 (Rv0644c), MmaA3 (Rv0643c), MmaA4 (Rv0642c), PcaA (Rv0470c), and CmaA2 (Rv0504c) [12,13,14]. By introducing cyclopropane rings and methyl modifications into the meromycoloyl chain, these enzymes generate alterations crucial for the pathogen virulence, pathogenicity, and persistence [15]. In addition, PDIM biosynthesis involves the S-adenosylmethionine-dependent methyltransferase Rv2952, which catalyzes the O-methylation of the third hydroxyl group of phthiotriol and phenolphthiotriol during the biosynthetic pathways of PDIM and phenolic glycolipid [16,17].

These findings, together with previous evidence showing that extractable lipids from ΔRv3787c negatively modulate CD4^+^ and CD8^+^ T cell responses to *M. tuberculosis*-stimulated immune cells from BCG-vaccinated healthy individuals [6], suggested a potential role for Rv3787c in lipid modifications. However, TLC analysis of extractable lipids of *M. tuberculosis* strains revealed no detectable alteration in the mycolic acids and PDIM profiles of the ΔRv3787c mutant.

These results do not exclude the possibility that Rv3787c contributes to subtle lipid modifications below the resolution of TLC, which could be explored using more sensitive lipidomic analyses.

A limitation of this study is that the predicted enzymatic activity of Rv3787c could not be experimentally confirmed. Rv3787c is a 308-amino-acid protein containing a predicted S-adenosylmethionine (SAM)-dependent methyltransferase domain spanning residues 15–285. However, recombinant expression of Rv3787c was unsuccessful, preventing biochemical validation of its predicted methyltransferase activity and limiting our ability to determine its potential role in the growth dynamics of *M. tuberculosis*. Further studies will be required to confirm its enzymatic activity, identify its potential substrate(s), and elucidate the molecular mechanisms underlying the growth phenotype associated with Rv3787c.

Given that SAM-dependent methyltransferases are required for resistance to detergent stress [18], we evaluated whether *Rv3787c* deletion affected detergent susceptibility and resistance to other stressors. The ΔRv3787c displayed no defects in growth under stress conditions, including detergent treatment or hydrogen peroxide challenge.

The most striking phenotype of ΔRv3787c was its accelerated recovery from stress, resuming growth more rapidly after the hypoxia phase and upon recovery from macrophages. This phenotype aligns with previous transposon mutagenesis studies, which classified *Rv3787c* mutants as having a “growth advantage” phenotype [4]. Similarly, Liu and collaborators [19] reported that *M. tuberculosis* mutants in the transcriptional regulator *resR* (*Rv1830*) recovered significantly faster from antibiotic treatment than the wild type, forming visible colonies earlier when transferred to drug-free media [19].

These observations, together with the phenotype of ΔRv3787c, suggest that mutations that reduce growth restraint following stress, such as antibiotic exposure, may provide a selective advantage in clinical isolates. This phenomenon could contribute to the emergence of strains with enhanced fitness in challenging environments.

In this regard, we examined the potential contribution of the A291V substitution identified in a high-fitness M-family isolate by complementing the ΔRv3787c mutant in the *M. tuberculosis* H37Rv background with an Rv3787c allele derived from a high-fitness isolate of the M family carrying this non-synonymous A291V substitution. However, no differences were observed in any of the phenotypes analyzed relative to ΔRv3787c complemented with the wild-type allele.

Under the stress conditions evaluated, our data do not indicate an effect of the A291V substitution on mycobacterial growth. Although this observation is consistent with AlphaFold structural predictions, suggesting that this substitution is unlikely to directly disrupt the predicted cofactor-binding site, we emphasize that these predictions are computational and do not constitute direct experimental evidence of the variant’s functional relevance. Specifically, we did not assay SAM binding, enzymatic activity, or protein stability experimentally, and our growth assays were restricted to a defined set of stress conditions. Consequently, an effect of A291V cannot be excluded: it may manifest under environmental conditions not examined here, or it may depend on the genetic background of the M strain, where epistatic interactions with additional polymorphisms could contribute to its high-fitness phenotype. Experimental validation, such as allelic exchange of A291V into an isogenic background, followed by growth and SAM-dependence assays across a broader range of conditions, will be required to establish the functional significance of this substitution.

In addition, RT-qPCR analysis showed substantially elevated Rv3787c transcript levels in the complemented strains due to the use of multicopy plasmids. Consequently, potential phenotypic differences between the alleles may have remained undetected or compensated for potential phenotypic differences associated with the different alleles.

Further studies using single-copy complementation strains, which would provide more physiologically relevant levels of Rv3787c expression, will be necessary to evaluate subtle or context-dependent effects of the A291V substitution. Therefore, the results presented here do not support a direct effect of A291V on the phenotypes examined, although they do not rule out a role for this variant in the enhanced fitness associated with M-family strains.

In addition, RT-qPCR analysis showed substantially elevated *Rv3787c* transcript levels in the complemented strains, a consequence of multicopy plasmid-based complementation. It is therefore possible that supraphysiological expression masked phenotypic differences between the alleles or alternatively compensated for deleterious effects of the A291V substitution. We emphasize that these explanations remain untested hypotheses, as we did not generate single-copy complementation strains. This limitation should be borne in mind when interpreting the absence of detectable differences, as overexpression-related artifacts cannot be excluded. Further studies using single-copy complementation systems, which would achieve more physiologically relevant levels of Rv3787c expression, will be required to resolve this question and to detect any subtle or context-dependent effects of the A291V substitution. Accordingly, the results presented here do not support a direct effect of A291V on the phenotypes examined; equally, however, they do not exclude a role for this variant in the enhanced fitness associated with M-family strains.

## 5. Conclusions

Although Rv3787c is dispensable for intracellular survival, our findings suggest that it contributes to the modulation of replication kinetics under environmental stress. The loss of genes associated with enhanced growth under laboratory in vitro or ex vivo conditions does not necessarily confer a global fitness advantage. Indeed, accelerated growth in nutrient-rich or otherwise permissive environments may entail a trade-off that becomes detrimental under physiologically relevant stresses. Genes such as Rv3787c may participate in metabolic adaptation, stress tolerance, persistence, or host–pathogen interactions; consequently, their absence could impair bacterial fitness in environments that more closely reflect those encountered during natural infection. Thus, the increased growth of ΔRv3787c observed in vitro may represent a context-dependent phenotype that does not translate into enhanced fitness in vivo. Characterization of the enzymatic activity of Rv3787c and its contribution to mycobacterial physiology will be important to clarify the mechanisms underlying this phenotype.

The conservation of the A291V mutation among high-fitness *M. tuberculosis* LAM strains [3] suggests that this substitution may contribute to adaptation within this lineage. However, our results indicate that the M allele of *Rv3787c* does not have a major or readily detectable role in the response of *M. tuberculosis* to the stress conditions examined here. Its contribution to fitness may instead be context-dependent, becoming apparent only under particular environmental challenges or during host infection. Assessing the A291V variant under conditions that more closely reproduce the host environment will help determine whether this mutation provides a selective advantage to the LAM family.

## Data Availability

The original contributions presented in this study are included in the article/Supplementary Materials. Further inquiries can be directed to the corresponding author.

## Supplementary Materials

The following supporting information can be downloaded at: https://www.mdpi.com/article/10.3390/biology15181666/s1, Supplementary Materials and Methods 1: Supplementary experimental procedures for cloning steps and colony PCR of mycobacterial colonies; Figure S1: Images of culture tubes during subculture of hypoxic cultures (starter) under aerated conditions; Figure S2: Transcript levels of *Rv3786c* were quantified by real-time RT-qPCR, using *sigA* as an endogenous reference and H37Rv as the calibrator sample. The Pair Wise Fixed Reallocation Randomization Test was used to assess fold-change differences between H37Rv and ΔRv3787c (ns: not significant); Figure S3; Structural localization of the A291V substitution in Rv3787c. Predicted AlphaFold structure of Rv3787c (*M. tuberculosis* H37Rv; AF-P9WFH3-F1). The position of residue A291, corresponding to the naturally occurring A291V substitution found in the high-fitness M strain, is highlighted in red. Predicted SAM-binding residues annotated in UniProt (131, 160, and 161) are highlighted in blue. The spatial separation between residue 291 and the putative SAM-binding region suggests that the A291V substitution is unlikely to directly affect cofactor recognition. DynaMut2 predicted a modest destabilizing effect of the substitution on protein stability (ΔΔG = −1.18 kcal/mol); Figure S4: Recovery of mycobacterial colonies from THP-1 cells at 4, 48, and 72 h post-infection following macrophage lysis. Following macrophage lysis, bacterial suspensions were diluted 1:100 at 4 h post-infection and 1:10 at 48 and 72 h post-infection and plated on solid medium. Images were acquired 16–18 days after plating and show the initially larger colony size of the ΔRv3787c strain compared with the wild-type H37Rv and complemented strains; Table S1: Primers used in this study; Table S2: Growth inhibition of *M. tuberculosis* strains under stress conditions.
