# Supplementary material for: Rv3787c Influences Growth Dynamics of Mycobacterium tuberculosis
Source: Biology (Basel). 2026 Sep 20;15(18):1666. doi: 10.3390/biology15181666 (PMC13604155; doi:10.3390/biology15181666)
Supplement: Supplementary file 1 [file biology-15-01666-s001.zip › biology-4490434-supplementary.pdf]

Supplementary Materials and Methods

1. Cloning steps:

M. tuberculosis H37Rv and *M. tuberculosis* Haarlem M strains were used as the genomic DNA source for the *Rv3787c* gene. *Escherichia coli* DH5α was employed for routine cloning and plasmid propagation. The shuttle expression vector pVV16, which carries a kanamycin resistance cassette (*aphA*), origins of replication for both *E. coli* (oriE) and mycobacteria (oriM), and the constitutive hsp60 promoter (Phsp60) upstream of a multiple cloning site (MCS), was used for complementation of mutant strain. *E. coli* strains were grown in Luria–Bertani (LB) broth or on LB agar at 37 °C. The *Rv3787c* coding sequence, excluding its native promoter, was amplified by PCR from *M. tuberculosis* strains genomic DNA using gene-specific primers (Table S1) engineered to incorporate NdeI (CATATG) and HindIII (AAGCTT) restriction sites at the 5′ and 3′ ends, respectively. The *Rv3787c* coding sequence was amplified by PCR under the conditions described below. The PCR product was separated by electrophoresis on a 0.8% (w/v) agarose gel, and the corresponding DNA band was excised and purified using the Monarch DNA Gel Extraction Kit (New England Biolabs, Ipswich, MA, USA), according to the manufacturer's instructions. The purified amplicon was initially cloned into the pGEM-T Easy vector (Promega, Madison, WI, USA) following the manufacturer's recommendations. Recombinant pGEM-T Easy plasmids carrying the *Rv3787c* coding sequence and the pVV16 plasmid were subsequently digested with NdeI and HindIII (New England Biolabs, Ipswich, MA, USA) in CutSmart® buffer at 37°C for 1–2 h. The digested pVV16 vector was treated with calf intestinal alkaline phosphatase (CIP; Promega, Madison, WI, USA) to minimize vector self-ligation. The digested DNA fragments were separated on a 0.8% agarose gel, and the bands corresponding to the *Rv3787c* insert and linearized pVV16 vector were excised using a sterile scalpel and purified with the Monarch DNA Gel Extraction Kit (New England Biolabs, Ipswich, MA, USA). The purified insert and vector were ligated at a 3:1 molar ratio using T4 DNA Ligase (New England Biolabs, Ipswich, MA, USA) and incubated overnight at 16°C. The ligation mixture was transformed into chemically competent *E. coli* DH5α cells by heat shock (30 min on ice, 42°C for 60 s, followed by 60 s on ice) and recovered in LB medium at 37°C for 30 min with shaking at 200 rpm. Transformants were selected on LB agar containing kanamycin (50 µg/mL) and incubated overnight at 37°C. Candidate colonies were screened by colony PCR, and positive clones were further analyzed by plasmid restriction digestion. The integrity and orientation of the *Rv3787c* insert were confirmed by Sanger sequencing using primers flanking the multiple cloning site (MCS). The validated pVV16-Rv3787c constructs (see map below) were subsequently purified and introduced into ΔRv3787c as described in the Materials and Methods.

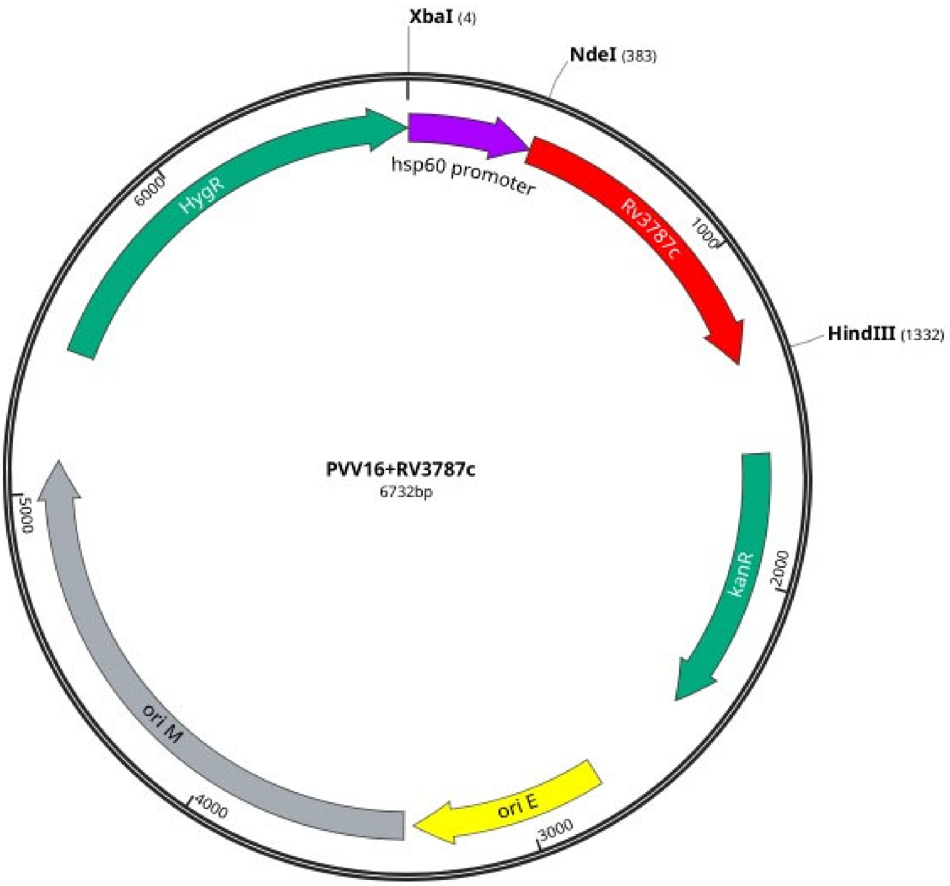

2. Colony PCR of mycobacterial colonies:

Individual mycobacterial colonies were picked using a sterile pipette tip and resuspended in 100 µL of nuclease-free water in a 1.5 mL tube. The suspension was subjected to thermal lysis at 90 °C for 45 min. The lysate was briefly centrifuged at 13,000 × g for 5 min to pellet cellular debris, and 1–2 µL of the supernatant was used directly as the DNA template. Each 25 µL PCR reaction was assembled using the SsoFast EvaGreen Supermix (BIO-RAD) mix, 0.2 µM each forward and reverse primer (Table S1). PCR amplification conditions. PCR amplification was carried out in a thermal cycler with the following program: an initial denaturation step at 98 °C for 3 min; 35 cycles of denaturation at 98 °C for 30 s, annealing at 60 °C for 30 s, and extension at 72 °C for 30 s; followed by a final extension at 72 °C for 7 min. The reactions were maintained at 16 °C until analysis. PCR products were resolved by electrophoresis on a 0.8% (w/v) agarose gel and visualized under UV light after staining with ethidium bromide or an equivalent nucleic acid stain.

Figure S1

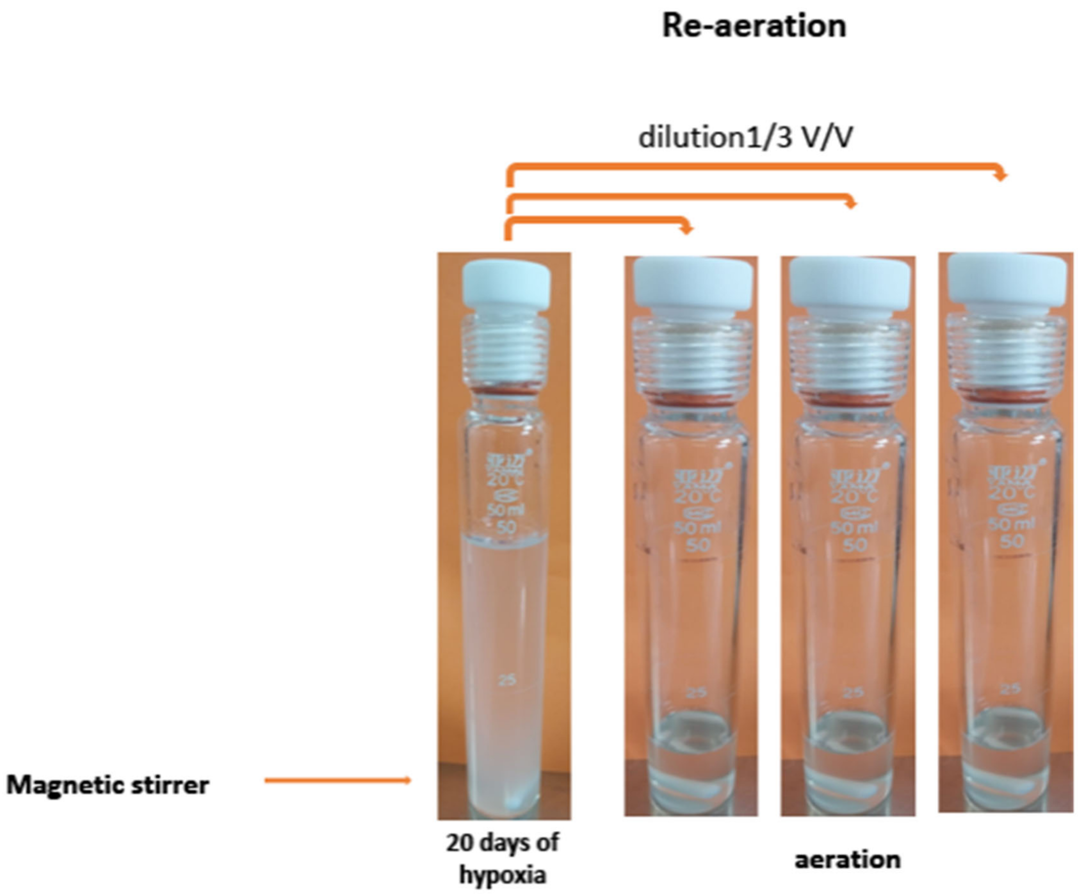
Re-aeration
dilution1/3 V/V
Magnetic stirrer
20 days of hypoxia
aeration

Figure S2

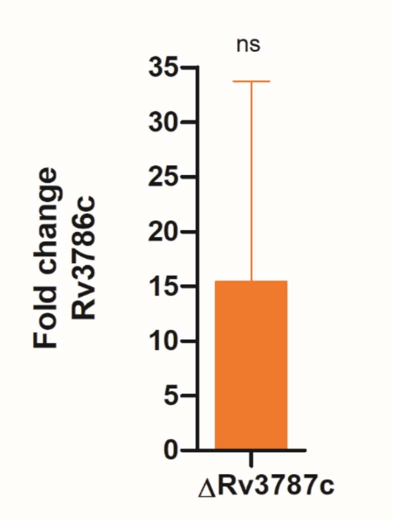

Figure S3

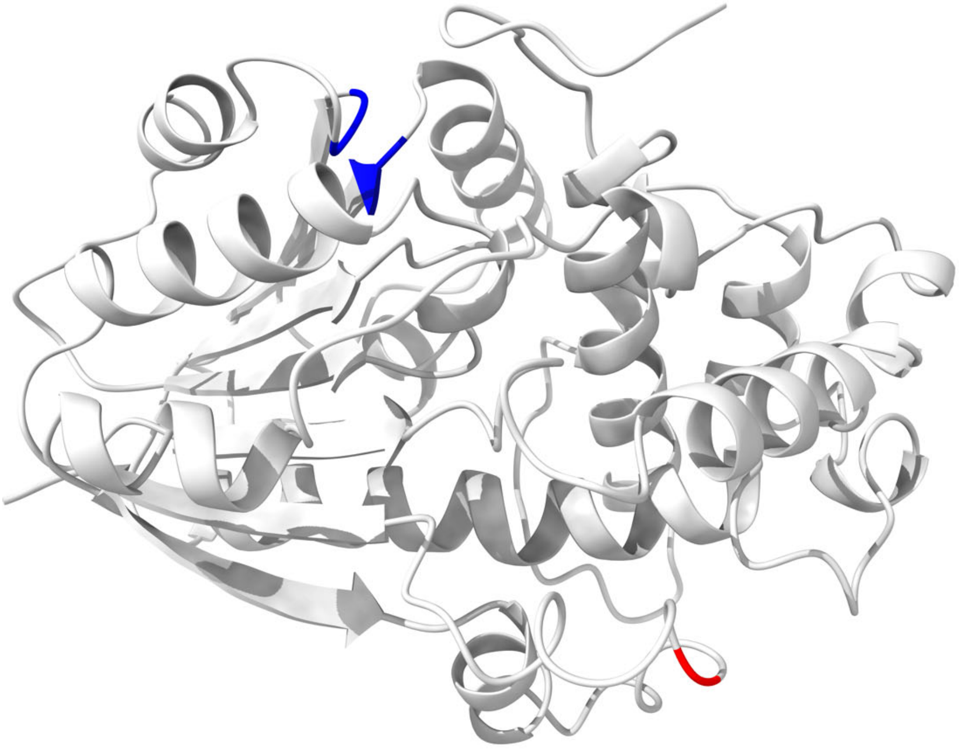

Figure S4

Time of infection (hs)

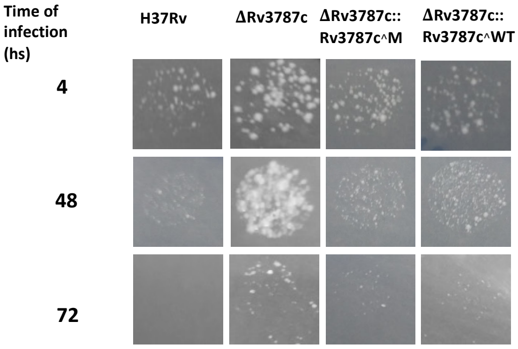

Table S1. Primers used in this study

Primer	Sequence 5´-3´	Used in:
up3787c_qPCR	cccgctacttcgatcagttc	RT-PCR, qPCR
low3787c_qPCR	gacctcgaacaccgtcgtat	RT-PCR, qPCR
up3786c_check	gctgatgtgtccgatggtgt	RT-PCR, qPCR
low3786_check	gtgctccaccctcaagatcc	RT-PCR, qPCR
up3787c	catatggtggcgcgtaccga	Complemented strain
low3787c	aagctttcaccccagaacaca	Complemented strain

Table S2. Growth inhibition of *M. tuberculosis* strains under stress conditions

Strain	SDS (10%)* (p=0.83)	H_2O_2 (6%)* (P=0.11)
H37Rv	19.66 (+/- 1.55)	34.4 (+/- 1.7)
ΔRv3787c	20.42 (+/- 1.63)	39.5 (+/- 4)

*surfaces of the inhibition halo (mm^2)
